# Supplementary material for: Chronic hypertension and perfusion deficits conjointly affect disease outcome after tPA treatment in a rodent model of thromboembolic stroke
Source: J Cereb Blood Flow Metab. 2025 Jan 22;45(5):831–41. doi: 10.1177/0271678X241310732 (PMC11755427; doi:10.1177/0271678X241310732)
Supplement: sj-pdf-1-jcb-10.1177_0271678X241310732 - Supplemental material for Chronic hypertension and perfusion deficits conjointly affect disease outcome after tPA treatment in a rodent model of thromboembolic stroke [file sj-pdf-1-jcb-10.1177_0271678X241310732.pdf]

Supplementary material

Franx, et al.

2024-08-02

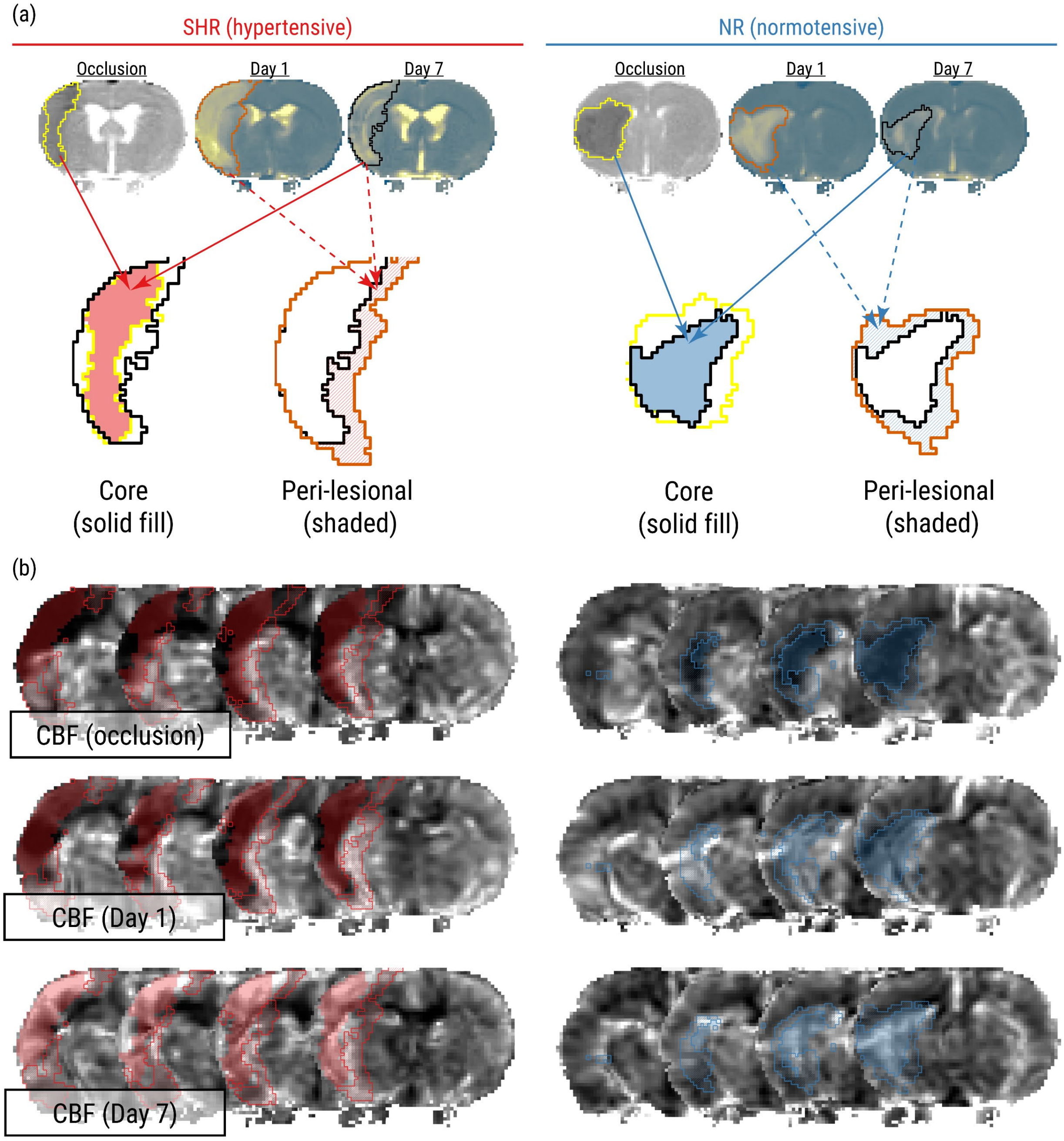

Supplementary Figure I (previous page). Visual aid to the region-of-interest (ROI) strategy and definitions. (a) Lesion masks were delineated from multiparametric data at different time points: the apparent diffusion coefficient (ADC) is sensitive to acute cytotoxic edema (during occlusion) and T_2-maps make ensuing vasogenic edema conspicuous (later time points on Day 1 and Day 7). Lesions at these time points were semi-automatically detected and are displayed here: cytotoxic edema during occlusion outlined in yellow, vasogenic edema at Day 1 outlined in orange, and the final infarct (also vasogenic edema) at Day 7 outlined in black. These lesion masks were combined to derive ROIs that satisfy the definitions described in the main text ("core" and "perilesional"). An example of this process is shown for both the hypertensive and normotensive rats, also shown in Figure 2. The "core" ROI is defined as initially ischemic tissue (during occlusion) that is ultimately infarcted on Day 7, therefore this ROI is the "intersection" between the masks obtained during occlusion and of the final lesion at Day 7 (both indicated with uninterrupted arrows), the new mask is the core ROI - marked by a solid fill. Similarly, the perilesional ROI is defined as tissue that shows signs of vasogenic edema on Day 1, but recovers by Day 7 (i.e., T_2 values are in the normal range). In simple terms, it can be said that the lesion mask from Day 7 is "subtracted" from the mask obtained at Day 1 (both indicated by dashed arrows); the new mask is the perilesional ROI - marked by a closed shading pattern for further illustration purposes in this Figure and in Figure 2 in the main text. (b) ROIs, obtained as described above, superimposed on CBF maps from these hypertensive and normotensive rats, at the different time points for multiple slices. Note: for every ROI at every time point, we extract only one value, an average from all voxels within that ROI, and these are normalized by fair control values from contralateral homologous areas (these areas are not shown). This operation produces data points shown in Figure 3(a) in the main text.

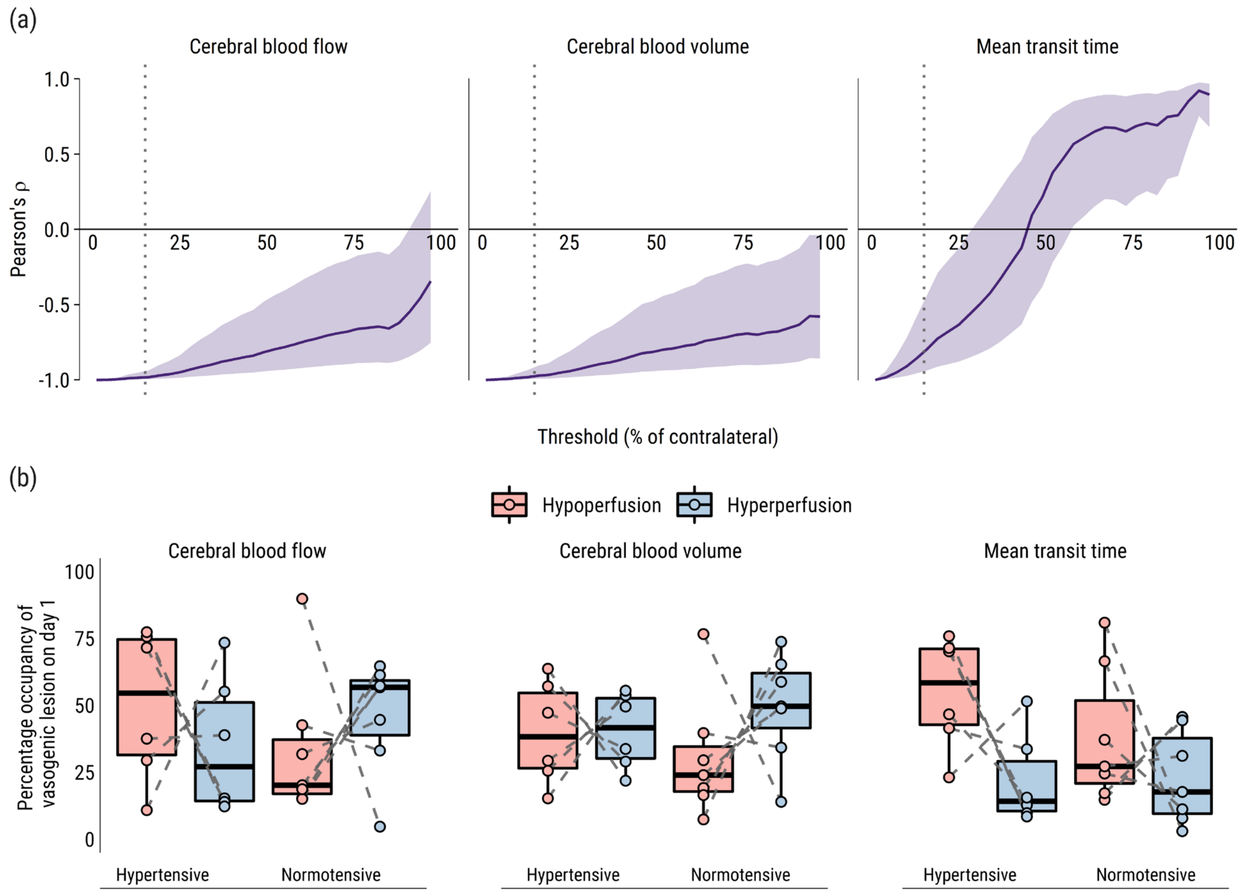

Supplementary Figure II. The correlation between reperfusion deficiencies in the vasogenic lesion (Day 1) and their definition. (a) Pearson's correlation coefficients between hypo- and hyperperfusion fractions, plotted as a function of threshold values. Shaded areas represent ±95% confidence interval. Dotted grey lines denote 15% threshold used for this analysis. (b) Percentages of hypo- and hyperperfused areas present within the vasogenic edematous lesion on day 1 (Figure 2(a)) using a 15% threshold. Dashed interconnected lines represent within-subject measurements, illustrating the origin of the strong negative correlation between hypo- and hyperperfusion.

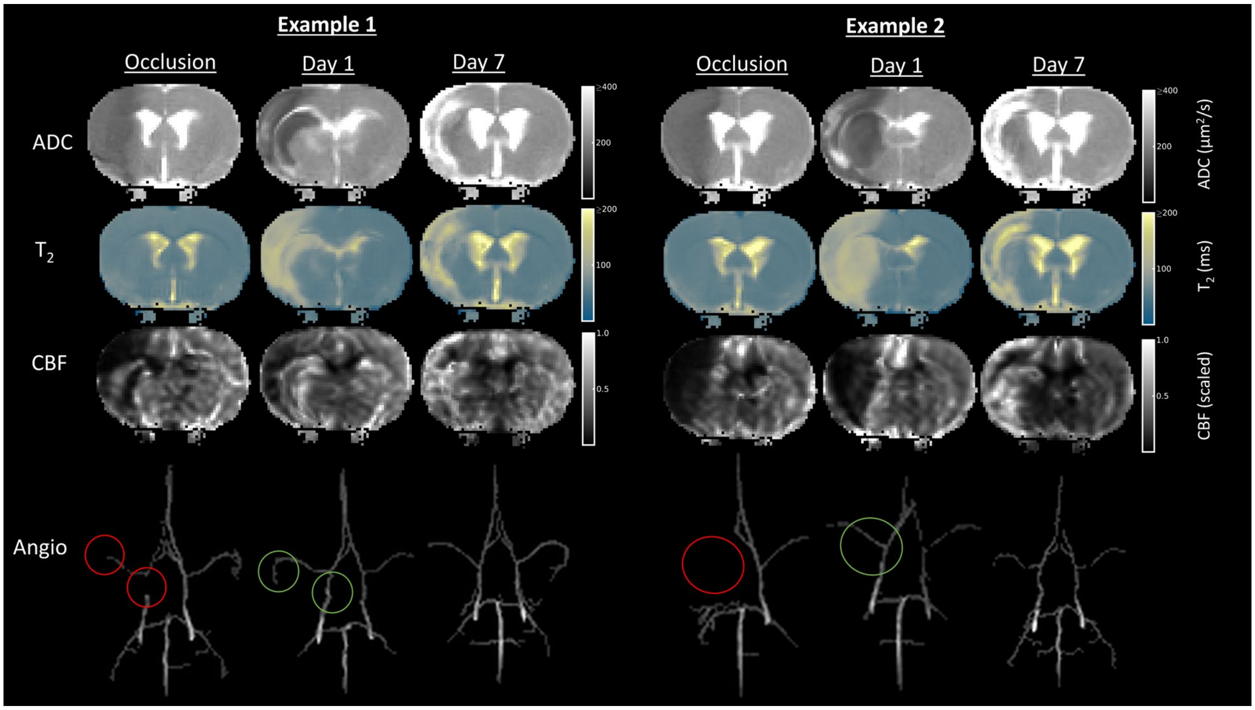

Supplementary Figure III. Serial coronal brain maps of ADC, T2 and CBF, and MR angiograms from two SHRs with recanalized feeding arteries but impaired reperfusion on day 1.

Supplementary Table I - RM-ANOVA – summary of linear mixed model perfusion analysis

CBF

Term	F	Df	Residual df	*p*
Strain	0	1	9.95	> 0.99
Time	***77.05***	***2***	***48.51***	***< 0.001***
ROI	***11.93***	***1***	***48.02***	***0.001***
Strain:Time	2.09	2	48.59	0.13
Strain:ROI	0.28	1	48.02	0.6
Time:ROI	***14.02***	***2***	***48.02***	***< 0.001***
Strain:Time:ROI	1.07	2	48.02	0.35

CBV

Term	F	Df	Residual df	*p*
Strain	0.18	1	9.93	0.68
Time	***45.56***	***2***	***48.68***	***< 0.001***
ROI	***6.19***	***1***	***48.04***	***0.016***
Strain:Time	0.29	2	48.79	0.75
Strain:ROI	0.73	1	48.04	0.4
Time:ROI	***17.19***	***2***	***48.04***	***< 0.001***
Strain:Time:ROI	1.21	2	48.04	0.31

MTT

Term	F	Df	Residual df	*p*
Strain	0.73	1	9.94	0.41
Time	***78.75***	***2***	***48.59***	***< 0.001***
ROI	***28.02***	***1***	***48.03***	***< 0.001***
Strain:Time	***4.23***	***2***	***48.68***	***0.02***
Strain:ROI	***8.06***	***1***	***48.03***	***0.007***
Time:ROI	2.55	2	48.03	0.089
Strain:Time:ROI	0.26	2	48.03	0.77

Supplementary Table II - Post-hoc analyses of cerebral perfusion indices before and after tPA

CBF

Strain	Time	contrast	Effect size	Estimate	SE	Resid df	t	*p*
SHR	***Occlusion***	***Perilesional - Core***	***2.09***	***45.07***	***12.45***	***48.02***	***3.62***	***0.004***
NR	Occlusion	Perilesional - Core	1.03	22.28	13.63	48.02	1.63	0.65
SHR	***Day 1***	***Perilesional - Core***	***2.07***	***44.67***	***12.45***	***48.02***	***3.59***	***0.005***
NR	***Day 1***	***Perilesional - Core***	***1.64***	***35.4***	***12.45***	***48.02***	***2.84***	***0.039***
SHR	Day 7	Perilesional - Core	-1.25	-26.93	12.45	48.02	-2.16	0.21
NR	Day 7	Perilesional - Core	-0.6	-12.96	12.45	48.02	-1.04	> 0.99

CBV

Strain	Time	contrast	Effect size	Estimate	SE	Resid df	t	*p*
SHR	***Occlusion***	***Perilesional - Core***	***2.02***	***0.44***	***0.13***	***48.04***	***3.49***	***0.006***
NR	Occlusion	Perilesional - Core	1.41	0.31	0.14	48.04	2.24	0.18
SHR	Day 1	Perilesional - Core	1.21	0.27	0.13	48.04	2.1	0.25
NR	***Day 1***	***Perilesional - Core***	***1.73***	***0.38***	***0.13***	***48.04***	***2.99***	***0.026***
SHR	***Day 7***	***Perilesional - Core***	***-1.95***	***-0.43***	***0.13***	***48.04***	***-3.37***	***0.009***
NR	Day 7	Perilesional - Core	-0.72	-0.16	0.13	48.04	-1.25	> 0.99

MTT (contrast = Strain)

Time	ROI	contrast	Effect size	Estimate	SE	Resid df	t	*p*
Occlusion	Core	SHR - NR	0.02	0	0.11	51.98	0.03	> 0.99
Day 1	***Core***	***SHR - NR***	***1.97***	***0.31***	***0.1***	***50.38***	***3.1***	***0.019***
Day 7	Core	SHR - NR	0.85	0.14	0.1	50.38	1.34	> 0.99
Occlusion	Perilesional	SHR - NR	-1.32	-0.21	0.11	51.98	-1.98	0.32
Day 1	Perilesional	SHR - NR	0.19	0.03	0.1	50.38	0.3	> 0.99
Day 7	Perilesional	SHR - NR	-0.1	-0.02	0.1	50.38	-0.15	> 0.99

MTT (contrast = ROI)

Strain	Time	contrast	Effect size	Estimate	SE	Resid df	t	*p*
SHR	***Occlusion***	***Perilesional - Core***	***-2.71***	***-0.43***	***0.09***	***48.03***	***-4.7***	***< 0.001***
NR	Occlusion	Perilesional - Core	-1.37	-0.22	0.1	48.03	-2.17	0.21
SHR	***Day 1***	***Perilesional - Core***	***-1.81***	***-0.29***	***0.09***	***48.03***	***-3.13***	***0.018***
NR	Day 1	Perilesional - Core	-0.02	0	0.09	48.03	-0.04	> 0.99
SHR	Day 7	Perilesional - Core	-1.32	-0.21	0.09	48.03	-2.29	0.16
NR	Day 7	Perilesional - Core	-0.37	-0.06	0.09	48.03	-0.64	> 0.99

Supplementary Table III - Interaction analyses - consecutive comparisons of contrasts before and after tPA

CBF

Time contrast	ROI contrast	Strain	Effect size	Estimate	SE	Resid df	t	*p*
Day 1 - Occlusion	Perilesional - Core	SHR	-0.02	-0.4	17.6	48.02	-0.02	> 0.99
Day 7 - Day 1	***Perilesional - Core***	***SHR***	***-3.32***	***-71.6***	***17.6***	***48.02***	***-4.07***	***< 0.001***
Day 1 - Occlusion	Perilesional - Core	NR	0.61	13.13	18.46	48.02	0.71	0.96
Day 7 - Day 1	***Perilesional - Core***	***NR***	***-2.24***	***-48.36***	***17.6***	***48.02***	***-2.75***	***0.017***

CBV

Time contrast	ROI contrast	Strain	Effect size	Estimate	SE	Resid df	t	*p*
Day 1 - Occlusion	Perilesional - Core	SHR	-0.8	-0.18	0.18	48.04	-0.99	0.66
Day 7 - Day 1	***Perilesional - Core***	***SHR***	***-3.16***	***-0.69***	***0.18***	***48.04***	***-3.87***	***< 0.001***
Day 1 - Occlusion	Perilesional - Core	NR	0.31	0.07	0.19	48.04	0.36	> 0.99
Day 7 - Day 1	***Perilesional - Core***	***NR***	***-2.45***	***-0.54***	***0.18***	***48.04***	***-3***	***0.009***

MTT

Strain contrast	ROI contrast	Time	Effect size	Estimate	SE	Resid df	t	*p*
NR - SHR	Perilesional - Core	Occlusion	1.34	0.21	0.14	48.03	1.57	0.12
NR - SHR	***Perilesional - Core***	***Day 1***	***1.78***	***0.28***	***0.13***	***48.03***	***2.19***	***0.034***
NR - SHR	Perilesional - Core	Day 7	0.95	0.15	0.13	48.03	1.16	0.25

Supplemental table IV: fractions of hypo- and hyperperfusion regressed against sensorimotor deficit score

	CBF		CBV		MTT	
Hypoperfusion (%)	0.0039		-0.0028		**0.0177***	
	(0.64)		(0.81)		**(0.04)**	
Hyperperfusion (%)		-0.0053		0.012		-0.028#
		(0.60)		(0.43)		(0.06)
Strain (hypertensive)	-0.59	-0.57	-0.66	-0.75	-0.66	-0.42
	(0.29)	(0.31)	(0.25)	(0.20)	(0.27)	(0.48)
Initial ischemic volume	0.0052	0.0050	0.0066#	0.0080*	0.0041	0.0042
	(0.12)	(0.14)	(0.06)	(0.03)	(0.18)	(0.15)
AIC	49.8	49.7	49.9	49.3	45.7	45.7
RMSE	1.39	1.38	1.42	1.38	1.12	1.03
Nagelkerke's R2	0.5	0.5	0.49	0.53	0.74	0.74
# p < 0.1, * p < 0.05, ** p < 0.01, *** p < 0.001						

Note: AIC = Akaike Information Criterion; RMSE = Root Mean Square Error; CBF = Cerebral Blood Flow; CBV = Cerebral Blood Volume; MTT = Mean Transit Time
